# A Study of the Etiology, Clinical Profile, and Diagnosis of Various Types of Central Nervous System Infections in a Tertiary Care Center

**DOI:** 10.7759/cureus.54250

**Published:** 2024-02-15

**Authors:** Shaiv Patel, Pranav Jhala, Himani Sharma

**Affiliations:** 1 Medicine, B. J. Medical College, Ahmedabad, IND; 2 Internal Medicine, Shri M. P. Shah Government Medical College, Jamnagar, IND; 3 Medicine, Government Medical College, Baroda, IND

**Keywords:** csf leukocytosis, headache, neck stiffness, meningitis, cns infection

## Abstract

Introduction

Infections affecting the central nervous system (CNS) can stem from various sources, including bacteria, viruses, and fungi, manifesting as conditions like meningitis, encephalitis, meningoencephalitis, and brain abscesses. Despite significant advancements in diagnosis and treatment, these infections continue to pose substantial risks to life. Several factors contribute to the causes of CNS infections. Demographic and geographic elements, the health status of individuals, their immune system's strength, the availability of diagnostic tools, and local prevention initiatives, all play pivotal roles. Consequently, the necessity of comprehensive local epidemiological data becomes undeniable as it guides the need for further studies and research. Understanding these factors is crucial for enhancing preventive measures and optimizing treatment strategies in tackling CNS infections.

Aims and objectives

This research aims to study the etiology and clinical features of different CNS infections among hospitalized patients and to diagnose cases of CNS infections based on laboratory and radiological investigations.

Material and methods

One hundred adults, seeking treatment for neurological impairments at a specialized tertiary care center in Gujarat, India, volunteered for this cross-sectional observational research. The study investigated the etiology, clinical profiles, and diagnoses of different CNS infections. It delved into the prevalence of these infections across age and sex categories while also observing mortality rates.

Results

In our research, we observed that bacterial causes were the most prevalent among CNS infections. Tubercular meningitis accounted for 36%, tuberculoma 14%, and pyogenic bacterial infections 23%. Following this, fungal infections emerged as the second most frequent, with mucormycosis at 9% and cryptococcus at 1%. Other less common CNS infections included viral encephalitis (4%), neurocysticercosis (3%), and brain abscess (1%). Middle-aged individuals between 41 and 60 years were most commonly affected (43%), followed by those aged 21-40 years (31%). Males accounted for a higher percentage of cases at 58%. Clinical symptoms revealed fever as the predominant feature (80%), with headaches following closely at 67%. Acute presentations were prevalent, representing 83% of cases, while neck stiffness was noted in 62% of patients. Most patients exhibited normal hemoglobin levels (96%) and a majority had a normal total leukocyte count (79%). Notably, 31% of the studied patients were identified as People Living With HIV (PLHIV). Out of 100 patients, 79 survived with appropriate treatment, resulting in a mortality of 21%.

Conclusion

The study identified various CNS infections, including bacterial (acute pyogenic meningitis, tubercular meningitis, tuberculoma, brain abscesses, and neurosyphilis), viral (viral meningitis and encephalitis), fungal (cryptococcal meningitis and CNS mucormycosis), and parasitic infections (neurocysticercosis and CNS toxoplasmosis). Tuberculous meningitis emerged as the most prevalent, followed by pyogenic meningitis. Clinical symptoms predominantly featured fever, headache, and altered sensorium, with less common occurrences of seizures, vomiting, weakness, and speech disturbances. Elevated CSF proteins and total leukocyte count were common findings in CSF analysis while consistent radiological observations included hypodensities in brain tissue and leptomeningeal enhancement.

## Introduction

Central nervous system (CNS) infections are complex and multifaceted, originating from a variety of sources, including bacteria, viruses, and fungi. These infections present themselves in several forms: meningitis, encephalitis, meningoencephalitis, and brain abscesses, each with unique neurological symptoms depending on the affected area. The diagnostic process for CNS infections is comprehensive, involving clinical assessments, laboratory analyses, and radiological evaluations. Despite advancements in diagnostic and treatment methods, CNS infections remain a significant cause of mortality and are a common reason for hospital admissions. While their prevalence is relatively lower in developed countries, CNS infections are still notably high in developing regions [1].

CNS infections include both meningeal and brain parenchymal infections, such as meningitis and encephalitis, which may occur independently or concurrently as meningoencephalitis. These infections can have bacterial, viral, fungal, or parasitic origins. Clinical presentations of the disease vary and can include meningitis, meningoencephalitis, or brain abscess. Common symptoms are fever, headache, nausea, vomiting, lethargy, myalgia, psychological and neurological deficits, neck pain and stiffness, photophobia, and general or focal seizures and paralysis. Meningitis, often identified by the clinical triad of fever, headache, and neck stiffness, takes various forms. Bacterial meningitis, for instance, involves pyogenic inflammation of the meninges and subarachnoid cerebrospinal fluid (CSF). Tuberculous meningitis (TBM) is particularly challenging in terms of treatment outcomes. Viral agents, such as herpes, mumps, measles, and HIV, can cause meningitis, with enteroviruses being the most common viral cause. Among fungal infections, cryptococcal meningitis is notably prevalent, especially in individuals with HIV [2-6].

A brain abscess can significantly impact its location by exerting a mass effect, displaying distinctive compression characteristics. This condition is not limited by age or racial differences. It occurs when infectious agents breach the protective barriers, namely, the skull, meninges, and blood-brain barrier. Such breaches can happen through various pathways, including hematogenous transmission, as seen in conditions such as tuberculosis (TB) and HIV meningitis [7]. Furthermore, certain viruses, such as arboviruses and respiratory viruses like herpes, rabies, and polio, spread through the neutropenic dissemination of the agent. Predisposing factors include otitis media, immune suppression, pneumonia, and sinusitis.

Investigating the etiology, clinical profiles, and diagnostic approaches of diverse CNS infections in a developing country context is crucial. It provides essential insights into the specific pathogens responsible for these infections, which is vital for developing tailored treatment strategies and region-specific vaccines. Additionally, understanding the varied clinical presentations is key to early and accurate diagnoses, leading to timely interventions that can significantly improve patient outcomes. In resource-limited settings, such studies are instrumental in guiding healthcare resource allocation and optimizing the use of available facilities and medications.

This study aims to explore the spectrum of clinical presentations associated with various CNS infections and establish accurate diagnoses through a comprehensive evaluation of clinical symptoms, laboratory findings, and radiological studies in hospitalized patients. This cross-sectional observational study was conducted to examine the etiology and clinical features of different CNS infections and to diagnose cases based on laboratory and radiological investigations.

## Materials and methods

Study design

This observational cross-sectional study was thoroughly reviewed and approved by the ethical board of the institute with IEC number MPSMC/91/02/2020. It involved a sample size of 100 patients admitted to a tertiary care center, all presenting signs and symptoms indicative of CNS infections.

Inclusion criteria

The study specifically included adult patients aged 18 years and older who were willing to participate. These participants presented with clinical symptoms and signs of CNS infection, along with CSF and radiological studies suggestive of an infectious etiology. The sample comprised patients who provided written informed consent. In patients with preserved sensorium patients themselves gave consent while in cases with lost sensorium, relatives were asked to give consent.

Exclusion criteria

Patients below 18 years of age and individuals affected by coronavirus disease 2019 (COVID-19) were excluded from the study. Additionally, patients who were not willing to participate in the study were also excluded.

Data collection and statistical analysis

The sample size calculation was based on the formula: (Zα)²pq/e², with a confidence level of 95%, Zα = 1.96, p = 40.8% (based on previous studies), q = 59.2, and e (margin of error) = 10%. This calculation resulted in an estimated 100 participants. The study spanned over a duration of 24 months, aiming to comprehensively investigate the various aspects of CNS infections within this patient sample.

The participants underwent comprehensive radiological examinations indicative of an infectious origin. This was supported by thorough history intake, meticulous clinical evaluations, and laboratory investigations, including CSF examination, all preceded by obtaining written informed consent.

A descriptive analysis was conducted in which the frequency and percentage of etiological factors’ prevalence in various age groups, along with the clinical features, CSF findings, and CT/MRI scan findings, were calculated.

## Results

**Figure 1:**
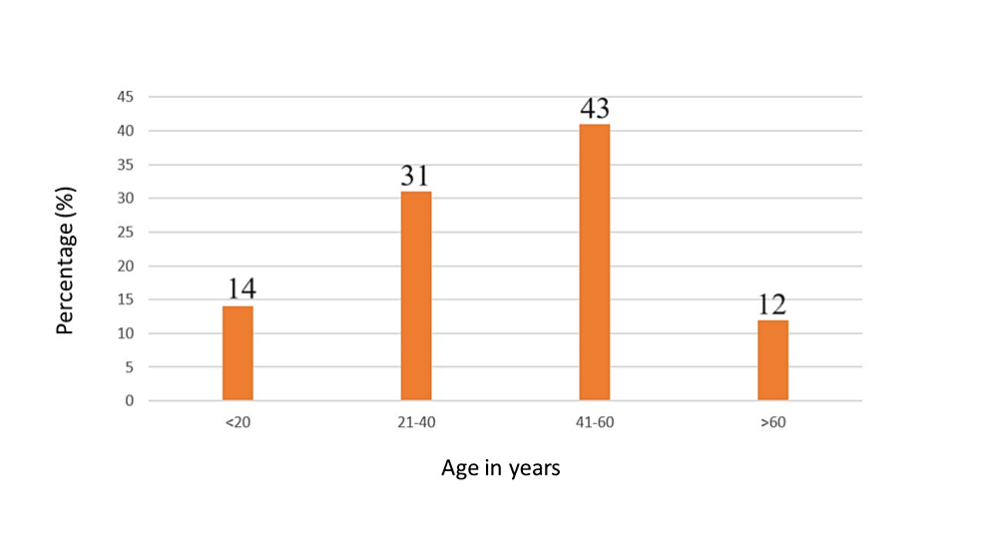
Frequency of CNS infections in different age groups

The frequency of CNS infections was highest in the 41 to 60 age group, accounting for 43% (n = 43), followed by the 21 to 40 years age group with 31% (n = 31). It was observed that CNS infections were less common in the older age group (>60 years), with a frequency of 12% (n = 12) (Figure 1). Since the study included patients older than 18 years of age, the frequency of CNS infections was lower in the age group below 20 years, with 14 out of 100 cases.

**Figure 2:**
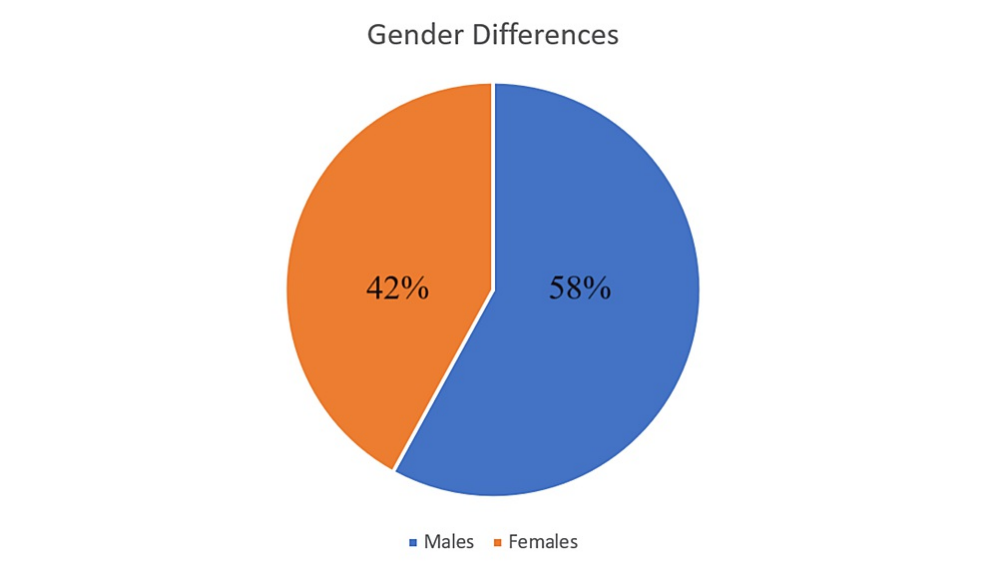
Gender differences in the prevalence of CNS infections

Our study found that CNS infections were more common in males, with a frequency of 58% (58 out of 100 cases). Although CNS infections occur in both sexes, they were slightly more prevalent in males (Figure 2).

**Figure 3:**
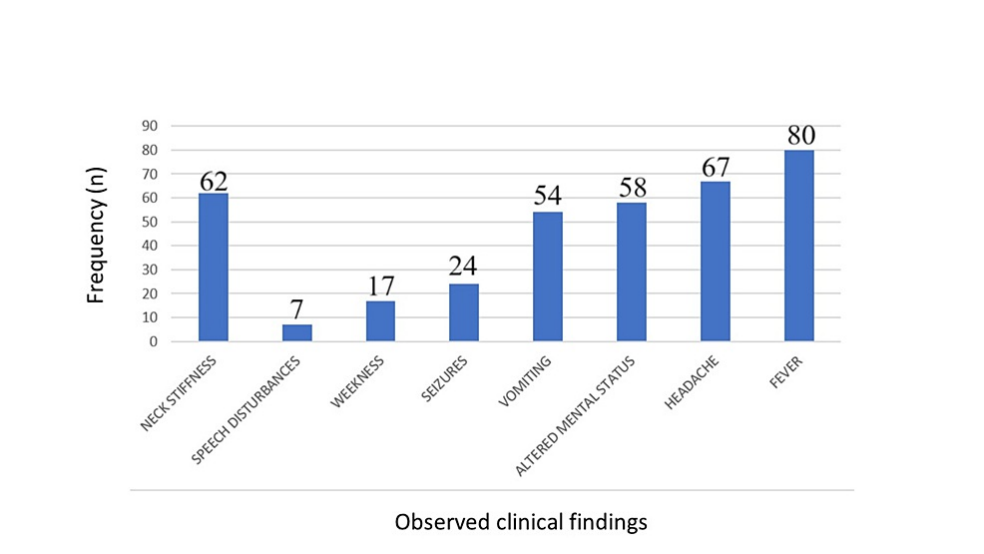
Frequency of various clinical features observed in patients with CNS infections

The clinical symptom evaluation visually represented in Figure 3 examines the occurrence and prevalence of various clinical presentations. Out of 100 patients with CNS infections, 31 (31%) were living with People Living With HIV (PLHIV) while the remaining 69 (69%) were not. Neck stiffness was observed in 62% (n = 62) of the patients.

**Figure 4:**
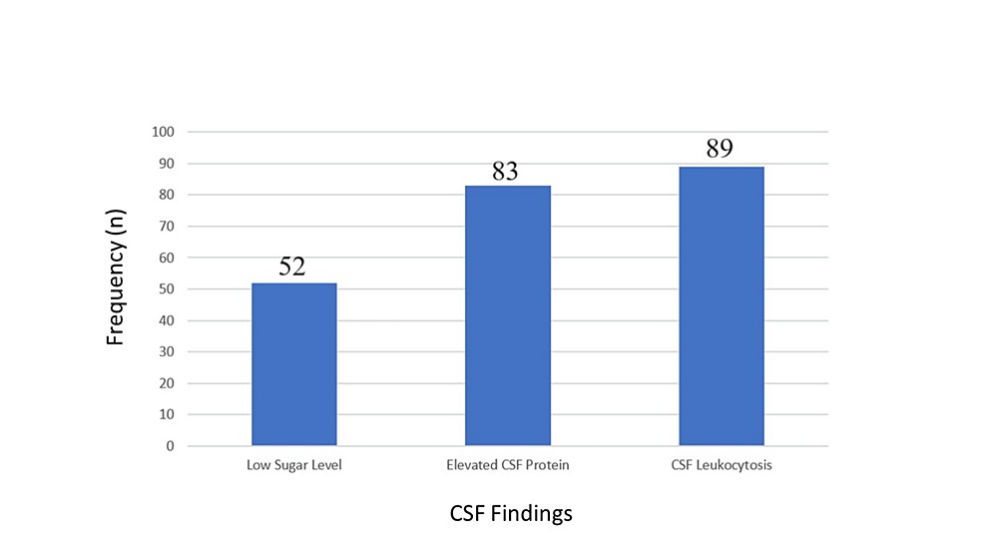
CSF findings in patients with CNS infections

Laboratory investigations of CSF are outlined in Figure 4, which includes a frequency chart for visual representation. Elevated protein levels in the CSF (i.e., >40 mg/dL) were found in 83% of patients (n = 83). Fifteen percent (n = 15) had normal protein levels (i.e., 20 to 40 mg/dL) while 2% (n = 2) had lower levels. CSF leukocytes, which may be neutrophils or lymphocytes, are considered significant when exceeding five cells per cumm. Our study found that 89% of the patients (n = 89) had significant CSF leukocytosis.

**Figure 5:**
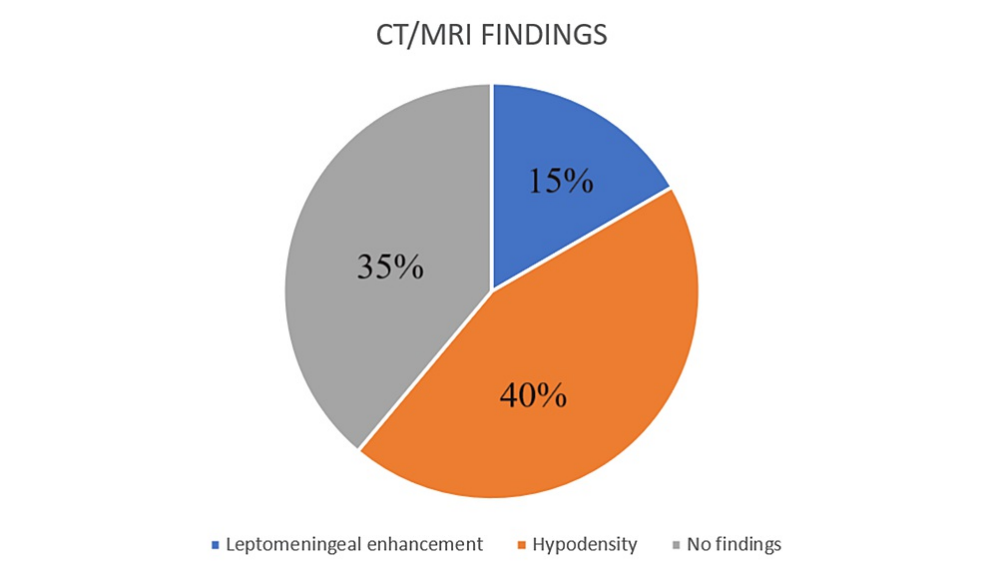
CT/MRI findings found in radiological investigations

CT/ MRI findings were present in 65% of patients. Leptomeningal enhancement was seen in 15% and there was hypodensity in 40%, which included space-occupying lesions, some ring-enhancing lesions, etc., which are highlighted in Figure 5.

In our study, we found that bacterial etiology is the most common; among those, 36% were tubercular meningitis, tuberculoma was 14%, and pyogenic bacterial infections were 23%. Fungal comes second with a frequency of mucormycosis of 9 followed by cryptococcus with a frequency of 1. Other less common CNS infections were viral encephalitis (4%), neurocysticercosis (3%), and brain abscess (1%), as detailed in Table 1.

**Table 1:** Observed etiology in the patients with CNS infections

Diagnosis	Frequency (n=100)	Percentage
Pyogenic bacterial meningitis	23	23%
Tuberculous meningitis	36	36%
Tuberculoma	14	14%
Neurosyphilis	1	1%
NCC	3	3%
Toxoplasma	5	5%
Cryptococcus	1	1%
Brain abscess	1	1%
Mucormycosis	9	9%
Viral encephalitis	4	4%
No diseases	3	3%
Total	100	100%

Of the 100 patients in the study, 79 survived with appropriate treatment, resulting in a mortality rate of 21%.

## Discussion

Despite significant advancements in diagnostic and treatment options, CNS infections remain serious health threats. The incidence of CNS infections studied was found to be highest in low-income countries, followed by middle-income and then high-income countries. It is essential to comprehend the diverse causes, clinical manifestations, and diagnostic approaches for CNS infections, especially in developing countries. This cross-sectional observational study aims to investigate the etiology and clinical characteristics of various CNS infections in hospitalized patients, using laboratory and radiological methods for diagnosis. The study focuses on examining prevalence across different age and gender groups and analyzing mortality rates, thereby providing a comprehensive understanding of CNS infections within this specific patient population.

As indicated in Figure 1, this study shows a higher prevalence of CNS infections in individuals aged 41-60 years, followed by those in the 21-40-year age group. Additionally, the data demonstrate a higher prevalence of these infections among males compared to females (Figure 2). In a study by Daniel Gams Massi et al. conducted in Cameroon, the average age of the patients was 42.6 ± 13.79 years, ranging from 16 to 84 years. The most affected age groups were those between 30-40 years (29.9%) and 40-50 years (28.4%), with males comprising 54.4% of the cases. Another multinational study across 25 countries reported an average patient age of 47.63 years (SD ± 19.8). In this study, 446 patients (17.3%) were elderly, averaging 74.58 years (SD ± 7.02). Among the patients with identified pathogens, 23.2% were over 65 years old, while 80.9% were under 65 years old [8].

In our study, 80% of the patients presented with fever (Figure 3). The second most common symptom was headache, followed by altered mental status. According to Erdem et al., common presenting symptoms included fever (94.5%), altered mental status (76.6%), and headache (70.6%). Cranial nerve palsies were frequent in CNS TB, with the most common being abducens (n = 13) and oculomotor (n = 7) palsies, affecting 27 patients in total [8]. Massi et al. noted that the most common neurological signs were headache (68.6%), impaired consciousness (44.1%), signs of meningeal irritation (38.7%), and epileptic seizures (36.3%). The main extra-neurological signs included an altered general state (93.1%), fever (84.8%), and vomiting (35.8%). On examination, most patients were vitally stable, with their sensorium being affected. Approximately 62% of patients exhibited neck stiffness with positive Kernig’s and Brudzinski’s signs. As mentioned above, only a few patients displayed clinical signs of raised ICT such as papilledema [9].

Diagnosis based on CSF studies, with lumbar puncture and CSF analysis being the mainstay in diagnosing different types of CNS infections, was a key feature of our study (Figure 4). The diagnosis of our study rested upon cerebrospinal fluid (CSF) analysis, with a focus on the predominant cell type - whether neutrophil or lymphocyte. The CSF analysis revealed a preponderance of neutrophils (>82%), a finding more commonly associated with bacterial infections while viral infections typically exhibit a greater prominence of lymphocytes (>85%). When only verified diagnoses were included, a higher concentration of CSF leukocytes was found in the category of acute bacterial meningitis (p = 0.0002) compared to other CNS infections. A significantly lower CSF/plasma glucose ratio was observed in cases with decreased intervals in bacterial infections.

As demonstrated in Figure 5, most meningeal infections were not detectable through radio imaging of the brain or spine. Only 39% of cases showed typical radiological findings indicative of CNS infections. The most common radiological appearances were hypodensity or space-occupying lesions, which were diagnosed as either tuberculoma or viral encephalitis. Another frequent finding in CT or MRI of the brain was leptomeningeal enhancement. This study indicates that some infections are diagnosed purely based on radiological evidence of pathology in the CNS, alongside similar clinical presentations with normal CSF studies. Conditions such as tuberculomas, CNS toxoplasmosis, neurocysticercosis, and brain abscesses are diagnosed through radiological investigations such as CT brain contrast studies and MRI of the brain.

In terms of WHO regions, Africa recorded the highest incidence of bacterial meningitis (65 cases per 100,000 people), NCC (650 per 100,000), and tuberculous spondylodiscitis (55 per 100,000). Southeast Asia had the highest incidence of intracranial abscess (49 per 100,000) while Europe reported the most cases of nontuberculous vertebral spondylodiscitis (5 per 100,000). Available case fatality data indicated the highest fatality rate for TBM/spondylodiscitis (21.1%) and the lowest for NCC (5.5%). The asymmetry in funnel plots, used to assess publication bias, suggests that these findings might underestimate the actual incidence of the diseases [10].

As shown in Table 1, this study emphasizes the predominance of CNS TB in India’s tertiary care centers, accounting for 36 out of 100 cases. The second most frequent infection is pyogenic meningitis, with other types of infections being relatively rare. In a study conducted by Devendra Kumar et al., which included 401 consecutive patients aged 12 years and older admitted to the medical emergency center, an etiological diagnosis was established in 365 patients (91%). Of these, 149 cases (40.8%) were confirmed through microbiological analysis. CNS TB was the most common diagnosis (51.5%), followed by viral meningoencephalitis (13.9%), community-acquired bacterial meningitis (9.7%), cryptococcal meningitis (6.2%), scrub typhus meningoencephalitis (1.7%), NCC (1.7%), and fungal brain abscess (1.7%). The most prevalent predisposing conditions for these infections were human immunodeficiency virus (11%) and diabetes mellitus (6.2%) [8].

Our study identified that 23% of patients were infected with bacteria causing pyogenic meningitis. According to Tyler et al., Hemophilus (H.) influenzae is occasionally found in non-vaccinated individuals. Nosocomial infections are commonly caused by S. pneumoniae, Staphylococcus aureus, Staphylococcus albus, and gram-negative bacilli. S. pneumoniae was the most prevalent species (58%), followed by GBS (18.1%), Neisseria meningitidis (13.9%), H. influenzae (6.7%), and Listeria monocytogenes (3.4%). Infectious meningitis can also be caused by viruses, fungi, and protozoa. Meningitis can have a noninfectious etiology such as cancer, medications, or inflammatory conditions [11].

Our study did not identify the organisms causing viral CNS infections, due to the prioritization of PCR for COVID-19 patients, but according to a study by Le Van Tan et al. in Vietnam, JEV was the most commonly detected virus (12%), followed by DENV (6.5%; CSF serology, n = 17 and CSF PCR, n = 2), HSV (6.5%), and EVs (2.7%) [12]. Our study found two manifestations of CNS TB: 36 patients presented with tubercular meningitis and 14 with parenchymal involvement as tuberculomas. Schaller et al. describe that CNS TB can manifest as a) diffuse TBM, b) localized tuberculoma, c) tuberculous abscess, or d) extradural and intradural spinal infections [7]. They highlight the clinical presentation, underlying pathology, and distinguishing features of TBM, which may lead to complications such as cranial nerve palsy, hydrocephalus, and infarction due to arteritis of the basal perforators.

Differential diagnoses include other infections (bacterial, viral, or fungal meningoencephalitis), malignant causes, or systemic inflammation with CNS involvement. Diagnosis and treatment are further complicated by factors such as HIV co-infection, multi-drug resistance, and TB-associated immune reconstitution inflammatory syndrome [7].

The limitations of this study include a limited sample size restricted to 100 individuals due to the impact of COVID-19 during the study period. Another limitation of this study is the exclusion of viral testing due to the prioritization of real-time reverse transcriptase-polymerase chain reaction (RT-PCR) for COVID-19 patients, leaving a gap in the characterization of respiratory illnesses based on viral pathogens utilizing PCR of CSF samples. Therefore, the pathogens causing the viral infection could not be identified. Consequently, future research could enhance its robustness by expanding to multiple centers or a larger population and performing specific tests to identify specific viral pathogens, hence, providing more in-depth insight and addressing the constraints inherent in this study.

## Conclusions

This study investigated a range of CNS infections caused by different pathogens, with certain types, such as TBM and pyogenic meningitis, being more prevalent than others. The infections predominantly affected individuals aged 41-60 years, with a higher incidence in males. Clinical symptoms varied, with fever being the most common, followed by headache and neck stiffness. Overall survival rates were observed following appropriate treatment, with a corresponding mortality rate. CSF analysis consistently revealed markers indicative of the inflammatory nature of these infections while radiological findings, including hypodensities in the brain parenchyma and leptomeningeal enhancements, were instrumental in the diagnostic process. In conclusion, this study underscores the diverse clinical presentations, diagnostic markers, and radiological features of CNS infections, emphasizing the necessity for a multifaceted approach to their management and diagnosis.
